# *Plasmodium* *falciparum* infection rates for some *Anopheles* spp. from Guinea-Bissau, West Africa

**DOI:** 10.12688/f1000research.5485.2

**Published:** 2014-11-05

**Authors:** Michelle R. Sanford, Anthony J. Cornel, Catelyn C. Nieman, Joao Dinis, Clare D. Marsden, Allison M. Weakley, Sarah Han, Amabelia Rodrigues, Gregory C. Lanzaro, Yoosook Lee

**Affiliations:** 1 Vector Genetics Laboratory, Department of Pathology, Microbiology, and Immunology, School of Veterinary Medicine, University of California, Davis, 95616, USA; 2 National Institute of Public Health (INASA), Bissau, Guinea-Bissau; 3 Department of Entomology and Nematology, University of California, Davis, 95616, USA; 4 Current Affiliation: Harris County Institute of Forensic Sciences, 1885 Old Spanish Trail, Houston, 77054, USA

## Abstract

Presence of *Plasmodium* *falciparum* circumsporozoite protein (CSP) was detected by enzyme linked immunosorbent assay (ELISA) in a sample of *Anopheles* *gambiae* s.s., *A. melas* and *A. pharoensis* collected in Guinea-Bissau during October and November 2009. The percentage of *P. falciparum *infected samples (10.2% overall; confidence interval (CI): 7.45-13.6%) was comparable to earlier studies from other sites in Guinea-Bissau (9.6-12.4%). The majority of the specimens collected were identified as *A*. *gambiae* which had an individual infection rate of 12.6 % (CI: 8.88-17.6) across collection sites. A small number of specimens of *A. coluzzii, A. coluzzii *x *A. gambiae *hybrids, *A*. *melas* and *A*. *pharoensis* were collected and had infection rates of 4.3% (CI:0.98-12.4), 4.1% (CI:0.35-14.5), 11.1% (CI:1.86-34.1) and 33.3% (CI:9.25-70.4) respectively. Despite being present in low numbers in indoor collections, the exophilic feeding behaviors of *A*. *melas* (N=18) and *A*. *pharoensis* (N=6) and high infection rates observed in this survey suggest *falciparum*-malaria transmission potential outside of the protection of bed nets.

## Introduction

Malaria is among the leading causes of childhood mortality in Guinea-Bissau, comprising 18% of mortality of children less than five years of age as of 2010 ( WHO, 2010). However, the human malaria incidence rate in Guinea Bissau varies considerably from year to year with a general decrease in recent years to about 3 children (<5 yrs of age) per thousand in some locations ( Ursing *et al.*, 2014). *Plasmodium falciparum* predominates, causing 98% cases, followed by a few cases of *Plasmodium malariae* and *Plasmodium ovale.* Mixed infections of *P*. *malariae*, and to a lesser extent *P*. *ovale*, have been recorded but appear to be rare and highly variable in both Guinea-Bissau ( Snounou *et al.*, 1993) and neighboring Senegal ( Fontenille *et al.*, 1997a; Fontenille *et al.*, 1997b).

Limited research has been conducted on the vectors and malaria parasite infection rates in Guinea-Bissau populations of *Anopheles* species in general and there is no data on comparative infection rates between *A. gambiae* and *A. coluzzii* and members of the *A. gambiae* complex. Variability is also high among the *Anopheles* spp. implicated as vectors in this region of West Africa in terms of both their temporal population dynamics as well as species composition among study sites ( Carnevale *et al.*, 2010; Fontenille *et al.*, 1997a; Jaenson *et al.*, 1994; Snounou *et al.*, 1993).

Here we present much needed data on *P*. *falciparum* infection of *Anopheles* spp. specimens collected from inside and around associated human habitations at eight sites in Guinea-Bissau ( Table 1).

**Table 1.** Sites, species and *Plasmodium falciparum* circumsporozoite protein (CSP) detection information from *Anopheles* spp. samples collected in Guinea-Bissau, October and November 2009. Numbers (#) indicate site locations on the map of Guinea-Bissau in Figure 1. All mosquitoes were collected indoors with a single exception; samples in Ponta Anabaca were opportunistically collected outside.

#	Site	*P. falciparum* infected	Uninfected	Total collected	Infection rate
***Anopheles coluzzii***					
1	Canjufa	0	1	1	0.0%
2	Bambadinca	2	16	18	11.1%
3	Antula	0	17	17	0.0%
4	Prabis	0	24	24	0.0%
5	Abu	1	7	8	12.5%
6	Brus	0	1	1	0.0%
8	Eticoga	0	1	1	0.0%
	**SUBTOTAL**	**3**	**67**	**70**	**4.3%**
***Anopheles gambiae***					
1	Canjufa	1	1	2	50.0%
2	Bambadinca	0	1	1	0.0%
3	Antula	13	63	76	17.1%
4	Prabis	3	50	53	5.7%
5	Abu	1	30	31	3.2%
6	Brus	0	5	5	0.0%
7	Ponta Anabaca	8	46	54	14.8%
8	Eticoga	3	5	8	37.5%
	**SUBTOTAL**	**29**	**201**	**230**	**12.6%**
***A. coluzzii***x***A. gambiae* hybrids**					
1	Canjufa	1	0	1	100.0%
3	Antula	1	26	27	3.7%
4	Prabis	0	14	14	0.0%
5	Abu	0	5	5	0.0%
8	Eticoga	0	2	2	0.0%
	**SUBTOTAL**	**2**	**47**	**49**	**4.1%**
***Anopheles melas***					
3	Antula	1	2	3	33.3%
4	Prabis	1	7	8	12.5%
5	Abu	0	2	2	0.0%
6	Brus	0	6	6	0.0%
8	Eticoga	0	1	1	0.0%
	**SUBTOTAL**	**2**	**16**	**18**	**11.1%**
***Anopheles pharoensis***					
2	Bambadinca	2	4	6	33.3%
	**Grand Total**	38	337	375	10.2%

## Method

**Figure 1.**
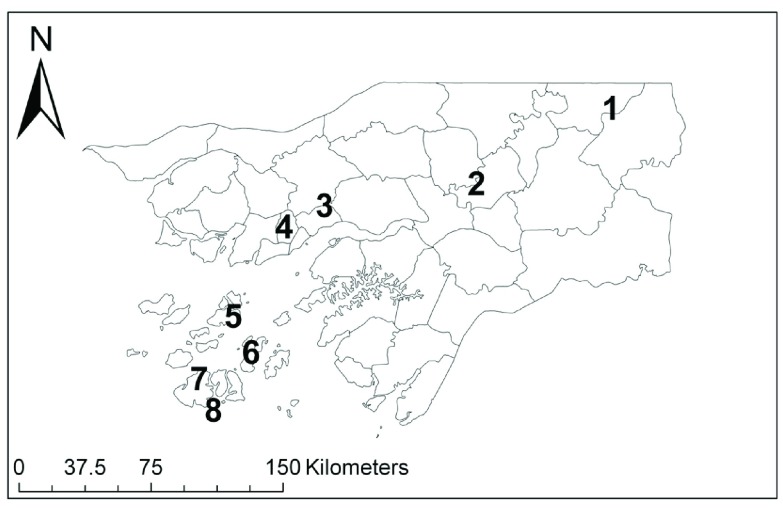
Collection sites in Guinea-Bissau. 1: Canjufa (12.43N, 14.13W), 2: Bambadinca (12.02N, 14.86W), 3: Antula (11.91N, 15.58W), 4: Prabis (11.80N, 15.74W), 5: Abu (11.46N, 15.91W), 6: Brus (11.23N, 15.88W), 7: Ponta Anabaca (11.18N, 16.14W) and 8: Eticoga (11.16N, 16.14W).

Mosquitoes were collected by mouth aspiration from both the island and inland areas of Guinea-Bissau ( Figure 1) in 2009 between October and November, which corresponds with the time of year previously observed to have the highest infection rate in *Anopheles* species ( Jaenson *et al.*, 1994). All mosquitoes were collected indoors with a single exception; samples in Ponta Anabaca were opportunistically collected outside while host-seeking at about 18:00hr. Each mosquito was dissected and the head and thorax were preserved in 100% ethanol for subsequent ELISA analysis. The remainder of each mosquito was preserved in 70% ethanol for genomic DNA extraction using the DNeasy extraction kit (Qiagen). Species determination of mosquitoes from the *A. gambiae* complex was made with the combination of species diagnostic assays ( Fanello *et al.*, 2002; Favia *et al.*, 2001; Santolamazza *et al.*, 2008; Scott *et al.*, 1993) and a divergence island SNP (DIS) genotyping assay ( Lee *et al.*, 2014a) while other species were identified by morphology.

For the Scott PCR ( Scott *et al.*, 1993) and the Fanello RFLP ( Fanello *et al.*, 2002), we used four primers (UN [5'-GTG TGG CCC TTC CTC GAT GT-3'], GA [5'-CTG GTT TGG TCG GCA CGT TT-3'], ME [5'-TGA CCA ACC CAC TCC CTT GA-3'] and AR [5'-AAG TGT CCT TCT CCA TCC TA-3']). We excluded QD primer ( Scott *et al.*, 1993) because our study site is well outside of the geographic range of this species (East Africa). A 25 µL PCR reaction containing 1X GeneAmp PCR Buffer (Applied Biosystems), 1mM MgCl _2_, 0.2mM of each dNTP, 0.12 µM of each primer and 0.05U AmpliTaq DNA polymerase (Applied Biosystems) was carried out for each individual. Scott PCR products were digested using Hha1 enzyme (New England Biosystems) following the protocol stated in ( Fanello *et al.*, 2002). Thermocycler conditions were 95°C for 5 min followed by thirty-five cycles of 95°C for 45 s, 50°C for 30 s and 72°C for 45 s, with a final elongation at 72°C for 7 min, and a 4°C hold.

For the Favia PCR ( Favia *et al.*, 2001), we used four primers (R5 [5'-GCC AAT CCG AGC TGA TAG CGC-3'], R3 [5'-CGA ATT CTA GGG AGC TCC AG-3'], Mopint [5'-GCC CCT TCC TCG ATG GCA T-3'] and B/S int [5'-ACC AAG ATG GTT CGT TGC-3']. A 25 µL PCR reaction containing 1X PCR Buffer (Applied Biosystems), 1.5mM MgCl _2_, 0.2mM of each dNTP, 0.2 µM of primer R5, 0.2 µM of primer R3, 0.16 µM of primer Mopint, 0.1 µM of primer B/S int and 0.02U DNA polymerase AmpliTaq (Applied Biosystems) was carried out for each individual. Thermocycler conditions were 95°C for 5 min followed by thirty-five cycles of 95°C for 30 s, 64°C for 30 s and 72°C for 30 s, with a final elongation at 72°C for 7 min, and a 4°C hold.

For the SINEX PCR ( Santolamazza *et al.*, 2008), we used S200 X6.1 forward [5'-TCG CCT TAG ACC TTG CGT TA-3'] and reverse [5'-CGC TTC AAG AAT TCG AGA TAC-3'] primers. A 25 µL PCR reaction containing 1X PCR Buffer (Applied Biosystems), 2mM MgCl _2_, 0.4mM of each dNTP, 0.2 µM of each primer and 0.1U DNA polymerase AmpliTaq (Applied Biosystems) was carried out for each individual. Thermocycler conditions were 95°C for 5 min followed by thirty-five cycles of 95°C for 30 s, 60°C for 30 s and 72°C for 30 s, with a final elongation at 72°C for 10 min, and a 4°C hold.

The resulting PCR products were analyzed on a Qiaxcel capillary electrophoresis instrument (Qiagen) using a DNA Screening Cartridge (Qiagen).

For DIS genotyping, we used Sequenom iPLEX Gold Genotyping Reagent Set (Catalog number: Sequenom 10158) and ran on the MassArray (Sequenom) mass spectrometer at the UC Davis Veterinary Genetics Laboratory. A mosquito was considered a hybrid if at least 5 out of 7 DIS on the X chromosome were in a heterozygous state.

*P. falciparum* infection was determined by enzyme linked immunosorbent assay (ELISA) of circumsporozoite protein (CSP) ( Burkot *et al.*, 1984; Wirtz *et al.*, 1987) from the head and thorax of mosquito specimens in an attempt to capture the parts of the mosquito that would indicate that they were infective. All chemicals except for substrate solutions (Item 5 on page 5 of the supplemental ELSA protocol document) were ordered from Sigma-Aldrich. Monoclonal antibodies (capture and conjugate) were obtained from Kirkegaard & Perry Laboratories. *P. falciparum* sporozoite protein for positive controls was ordered from the Centers for Disease Control and Prevention (CDC). We followed the Sporozoite ELISA directions provided by the CDC (Sep, 2009 version) with a few modifications (see supplemental document for the modified ELISA protocol). Samples were considered positive if absorbance values were three or more standard deviations from the negative control samples (99% CI) on each ELISA plate ( Beier *et al.*, 1987; De Arruda *et al.*, 2004).

The results of the ELISA were analyzed for both CSP concentration, adjusted for plate-to-plate variation, with an analysis of variance and for a binary outcome using a χ ^2^ test implemented in SPSS 16.0 ( SPSS, 2007). The data were analyzed for differences between species and among collection sites, using a G-test implemented in Deducer library under R software ( http://www.r-project.org/). A Confidence interval (CI) was calculated using adjusted Wald confidence intervals using an online calculator ( www.measuringu.com/wald.htm). Mosquito species. *P. falciparum* infection state and CSP concentration for each individual are provided in Dataset 1.

## Results & discussion

ELISA results identifying Plasmodium falciparum infection status in Anopheles spp. collected in Guinea-Bissau Mosquitoes were collected at eight different sites in Guinea-Bissau between October and November 2009. All mosquitoes were collected indoors except at Ponta Anabaca which were collected outdoors. See associated article for methods.

Four species, *A. coluzzii, A. gambiae*, *A. melas*, *A. pharoensis* were collected during sampling. A number of *A*. *coluzzii* x *A*. *gambiae* hybrids were also collected. All mosquitoes were collected indoors with a single exception; samples in Ponta Anabaca were opportunistically collected outside of a human habitation while apparently host-seeking immediately after sunset at about 18:00 hr, which is earlier than reported observations for members of the *A. gambiae* complex in The Gambia (West Africa) ( Lindsay *et al.*, 1989; Snow *et al.*, 1988). All species were collected at multiple sites except *A. pharoensis,* which was only collected at the more inland site of Bambadinca. *A. pharoensis* is not generally considered a significant vector in West Africa but the distribution observed in this study matches the previously observed pattern in Senegal ( Carrara *et al.*, 1990). *Anopheles arabiensis* was absent from collections.

No significant differences were observed for CSP concentration or in the analysis of positive samples with χ ^2^. This is probably due to the variation in the distribution of vector species and *P*. *falciparum* in the environment at the time of sampling. Table 1 presents CSP rate data and the total number of each individual species collected at each site.

The percentage of *P*. *falciparum* positive samples from members of the *A. gambiae* species complex observed in this study (overall 10.2%; CI:7.45-13.6%) were similar to earlier studies in other regions in Guinea-Bissau (12.0% ( Snounou *et al.*, 1993) and 9.6–12.4% ( Jaenson *et al.*, 1994)). The overall CSP positive rate for *A. gambiae* was 12.6% (CI 8.88-17.6%) and 11.1% (CI:1.86-34.1%) for *A. melas*. Previously published CSP positive rates for *A. gambiae* s.s. (= *A. gambiae* and *A. coluzzii*) range between 2.24% in Guinea ( Carnevale *et al.*, 2010) to 9.6% in Guinea-Bissau ( Jaenson *et al.*, 1994). Earlier studies when individual species within the *A. gambiae* complex were not identified, infection rate of *A. gambiae* s.l. ranged from as high of 17.73% in the eastern regions of The Gambia ( Thomson *et al.*, 1994) to 12% in Guinea-Bissau ( Jaenson *et al.*, 1994; Snounou *et al.*, 1993). The CSP positive rate was significantly higher in *A. gambiae* (12.6%) than *A. coluzzii* (4.3%) (Wilcoxon rank sum test P-value=0.0384). This is consistent with the earlier study in Senegal ( Ndiath *et al.*, 2011) but differs from a recent survey conducted in Mali ( Fryxell *et al.*, 2012). The study site in Senegal located in the village of Dielmo (13°43'N, 16°24'W) ( Ndiath *et al.*, 2012) was geographically closer (200km) than Mali sites (>800km) to our collection sites in Guinea-Bissau. The Senegal study site at Dielmo and nine of our study sites were proximal (<50km) to the Atlantic Ocean, while Mali is a land-locked country at least 500km away from the Atlantic Ocean. Therefore, the discrepancy among studies may be due to climatic and environmental pressure on the different genetic backgrounds of *A. gambiae* observed in this area of West Africa ( Lee *et al.*, 2013). More robust sampling over a larger number of collection sites would help in confirming this trend.

In this study, a few *A. pharoensis* (N=6) were collected, half of which were CSP positive. Other studies in this region of West Africa have found that *A. funestus* and *A. arabiensis* may also be important vector species at different times in nearby Senegal ( Fontenille *et al.*, 1997a; Fontenille *et al.*, 1997b). *A. arabiensis* was not collected in our study while a small number (N<10) of *A. funestus* were observed but not collected.

Recent studies on the prevalence of malaria parasites in humans have suggested that infection rates in Guinea-Bissau may be in decline due to widespread use of effective treatment and insecticide treated bed nets (ITNs and long lasting insecticide treated bed nets, LLINs) by the most high-risk groups ( Rodrigues *et al.*, 2008; Ursing *et al.*, 2014). The malaria parasite life cycle is complicated and may not directly relate to the prevalence of human cases but it is possible that the lack of data during periods of political unrest has concealed a more stochastic pattern than was previously observed in Guinea-Bissau ( Ursing *et al.*, 2014).

Outdoor mosquito collection was not the focus of this survey and was only made at Ponta Anabaca Hotel grounds when we fortuitously noted mosquitoes biting. Consequently no general comments about the degree of exophily of *A. gambiae* in Guinea-Bissau can be made. However, evidence of exophily by the major malaria vector *A. gambiae* in this study and by others in West Africa ( Reddy *et al.*, 2011; Tchouassi *et al.*, 2012) raises the concern of the long term effectiveness of Indoor Residual Spraying (IRS) and Long lasting Insecticide-treated Nets (LLINs) in reducing outdoor transmission of malaria especially before bedtime and by people sleeping outdoors. The relatively high infection rate of 11.1% of *A. melas* in Guinea-Bissau together with its tendencies to be both endophilic and exophilic and have a high human blood index ( Sharp *et al.*, 2007; Tuno *et al.*, 2010) make the species a significant vector, which may also be hard to control by reliance on ITNs and LLINs.

The high CSP rate of 33.3% in the 6 indoor collected *A. pharoensis* might implicate a significant role in malaria transmission in drier inland Guinea Bissau, however this should be viewed with caution due the small sample size. This contrasts the recent finding in Mozambique where none of the 4390 *A. pharoensis* samples were positive for CSP-ELISA ( Charlwood *et al.*, 2013). Very low infection rates and absence of malarial parasites, traditionally found in West and Central African populations of *A. pharoensis* has always led to the conclusion that this mosquito plays little role in malaria transmission despite its anthropophilic habits and that it can be easily experimentally infected ( De Meillon, 1947; Ndiath *et al.*, 2012; Tchouassi *et al.*, 2012). In drier Sahel regions of Africa where the major vectors of malaria are absent or very rare and irrigated rice and other crop lands are increasing, *A. pharoensis* is considered more important at maintaining low levels of malaria ( Kerah-Hinzoumbé *et al.*, 2009; Kibret *et al.*, 2010). More rigorous sampling effort and infection confirmation using multiple approaches (ELSA + SNP genotyping assays ( Lee *et al.*, 2014b) will be required to confirm the definitive role of this species in malaria transmission in this region.

## Data availability

figshare: ELISA results identifying *Plasmodium falciparum* infection status in *Anopheles* spp. collected in Guinea-Bissau. doi: 10.6084/m9.figshare.1200058 ( Sanford *et al.*, 2014).

## Supplementary materials

**Sporozoite ELISA Directions**. Click here to access the supplement.
