# Supplementary material for: Plasmodium falciparum infection rates for some Anopheles spp. from Guinea-Bissau, West Africa
Source: F1000Res. 2014 Nov 5;3:243. Originally published 2014 Oct 14. [Version 2] doi: 10.12688/f1000research.5485.2 (PMC4215749; doi:10.12688/f1000research.5485.2)
Supplement: Peer review history

# Peer review: Referee response for version 1

**DOI:** 10.5256/f1000research.5855.r6412

**Published:** 2014-10-23

**Authors:** Guido Favia

**Affiliations:** 1 School of Bioscience and Veterinary Medicine, University of Camerino, Camerino, Italy

This “observation article” is very well written in a format that is accessible to both general and specialist audience. It describes some novel observations about malaria infection rates in different vector species in Guinea-Bissau. In particular it reports the some how unexpected high infection rates in *Anopheles melas* and *A. pharoensis*, thus suggesting *Plasmodium falciparum*-malaria transmission potential outside of protection (i.e. bed nets).

Details about the circumstances of the finding and evidence of the observation are properly provided. The manuscript appropriately cites relevant bibliography in the field. Figures and tables are informative and helpful. Methods section is well organized and nicely descriptive.

As observational article it looks perfectly adequate to the journal purpose.

I have only a very minor concern: in the Introduction (line 8) *Plasmodium malaria *should be re-written as *Plasmodium malariae*.

I have read this submission. I believe that I have an appropriate level of expertise to confirm that it is of an acceptable scientific standard.

# Peer review: Referee response for version 1

**DOI:** 10.5256/f1000research.5855.r6413

**Published:** 2014-10-22

**Authors:** J Derek Charlwood

**Affiliations:** 1 London School of Hygiene & Tropical Medicine, London, UK

This paper provides information on sporozoite rates from a relatively unstudied area the mainland and islands of Guinea Bissau. It gives the impression that it is a spin off from another study that perhaps aimed at characterizing the genetics of populations of *Anopheles gambiae* from the mainland and the islands, perhaps for future genetic control efforts.  Given the widespread use of mosquito bednets rates are exceptionally high and not, apparently different from rates recorded earlier. The authors do make the comment that this might be the result of civil strife in Guinea-Bissau but whatever the cause this is disquieting and implies that the gains in reduction of malaria are going to be at best temporary.

The data are presented without confidence intervals but these should be added. Given the relatively small numbers involved either adjusted Wald confidence intervals (that can easily be calculated using the site www.measuringu.com/ **wald**.htm or a routine in R) can, I think, be used. (But since I am signing this review everyone should know that my statistical abilities are limited!

The kind of collection undertaken needs to be explained in more detail. Were the mosquitoes collected resting or were they landing collections? I do not really want to be the person raising this issue but something on ethics should be included somewhere. (My own thoughts on ethics in general is that if the rule of ‘first do no harm and second maybe do some good’ is followed then a study – that may include even *ad hoc* landing collections – is not unethical.) This is especially important if the collections were landing collections.

To avoid possible misunderstanding, the sentence ‘Four species were collected during sampling; *A. coluzzii, A. gambiae*, *A. melas*, *A. pharoensis* and *A*. *coluzzi* x *A*. *gambiae* hybrids were observed ‘ should be rewritten (since it could be misinterpreted) perhaps as two sentences: ‘Four species, *A. coluzzii, A. gambiae*, *A. melas*, *A. pharoensis* were collected during sampling. A number of *A*. *coluzzi* x *A*. *gambiae* hybrids were also collected’

There are a number of small errors in the paper that need to be rectified. For example in the last paragraph they state ‘33% of the 4 *Anopheles pharoensis* collected indoors when they either mean 33% of the six Anopheles pharoensis collected or 50% of the four collected indoors. With regard to this species it may be worth pointing out that in Mozambique none of the 4390 tested were positive for sporozoites ( Charlwood *et al.,* 2013) but at the same time in Ghana, ( Dzodzomenyo *et al.*, 1999) found that two of three specimens of *An. pharoensis *examined were infected (with Bancrotian filariasis) and one of these was infectious.  Given the possibility of false positives among primarily zoophilic anophelines (that may also include *An. melas*) and given that the authors have access to a sophisticated laboratory it is a shame that they did not run a PCR on the sporozoite positive specimens to ensure that they were indeed human malarias.

I have read this submission. I believe that I have an appropriate level of expertise to confirm that it is of an acceptable scientific standard.
